# SearchSmallRNA: a graphical interface tool for the assemblage of viral genomes using small RNA libraries data

**DOI:** 10.1186/1743-422X-11-45

**Published:** 2014-03-07

**Authors:** Roberto RS de Andrade, Maite FS Vaslin

**Affiliations:** 1 Departamento de Virologia, Instituto de Microbiologia, Universidade Federal do Rio de Janeiro (UFRJ), 21941-590 Rio de Janeiro, RJ, Brasil

## Abstract

**Background:**

Next-generation parallel sequencing (NGS) allows the identification of viral pathogens by sequencing the small RNAs of infected hosts. Thus, viral genomes may be assembled from host immune response products without prior virus enrichment, amplification or purification. However, mapping of the vast information obtained presents a bioinformatics challenge.

**Methods:**

In order to by pass the need of line command and basic bioinformatics knowledge, we develop a mapping software with a graphical interface to the assemblage of viral genomes from small RNA dataset obtained by NGS. SearchSmallRNA was developed in JAVA language version 7 using NetBeans IDE 7.1 software. The program also allows the analysis of the viral small interfering RNAs (vsRNAs) profile; providing an overview of the size distribution and other features of the vsRNAs produced in infected cells.

**Results:**

The program performs comparisons between each read sequenced present in a library and a chosen reference genome. Reads showing Hamming distances smaller or equal to an allowed mismatched will be selected as positives and used to the assemblage of a long nucleotide genome sequence. In order to validate the software, distinct analysis using NGS dataset obtained from HIV and two plant viruses were used to reconstruct viral whole genomes.

**Conclusions:**

SearchSmallRNA program was able to reconstructed viral genomes using NGS of small RNA dataset with high degree of reliability so it will be a valuable tool for viruses sequencing and discovery. It is accessible and free to all research communities and has the advantage to have an easy-to-use graphical interface.

**Availability and implementation:**

SearchSmallRNA was written in Java and is freely available at http://www.microbiologia.ufrj.br/ssrna/.

## Background

During viral infection, antiviral silencing host pathways known as RNA interference (RNAi) can be triggered by the presence of viral double-stranded RNAs (dsRNA). These dsRNA structures are recognized and processed into vsRNAs that vary in length from 21 to 24 nucleotides. The vsRNA accumulates in the cytoplasm and may be even amplified as secondary vsRNAs in plant and invertebrate hosts [1]. Kreuze *et al*. [2] reported the first identification of novel viruses and the sequencing of an entire viral genome using high-throughput parallel sequencing of small RNAs (sRNA) from diseased, as well as symptomless plants. This represented a novel approach in the identification of known viral pathogens that occur at extremely low titers and of novel viruses that required no prior knowledge of the virus. Recently, others researchers have used this technique to sequence viral genomes. For example, Wu *et al.*[3] examined contigs assembled from published small RNA libraries, and discovered five previously undescribed viruses from cultured Drosophila cells and adult mosquitoes, including three with a positive-strand RNA genome and two with dsRNA genomes.

The most commonly used software for viral identification by assembly of NGS data is Velvet [4]. Velvet can span parts of the viral genomes by the assembly of vsRNA into contigs. However, the contigs generated by this tool do not cover all the virus genome, Information of hundred to thousand vsRNAs is lost from the dataset. Thus, the sequenced virus genome may contain uncovered regions or gaps. One approach to solve this limitation is to use command-line programs and small computer algorithms to map the viral genome from the complete small RNA dataset. Therefore, researchers with bioinformatics expertise have become indispensable for virus genome mapping from NGS libraries data. To bypass the need of this kind of expertise or knowledge, and to permit the mapping and assemblage of viral genomes using NGS datasets, we developed SearchSmallRNA, an easy-to-use software that does not require any knowledge of programming languages and can be used by all researchers.

SearchSmallRNA is the first mapping program that exclusively uses sRNA (host sRNA?+?vsRNAs) data sets to assemble viral genome. In addition to mapping the viral genome, it presents the data graphically and shows statistical analyses directly from the large and complex sets of small RNA sequences from infected hosts, fitting together tools that were previously available only as independent scripts or softwares.

## Methods

### Software

SearchSmallRNA was developed in JAVA language version 7 using NetBeans IDE 7.1 software. Biojava3-core-3.0.2.jar and Biojava3-alignment-3.0.2.jar packages were used as additional tool, but the search engine doesn’t use traditional alignment to find similar strings. It searches reads (small DNA strings with 18-24nt length) in a reference genome comparing their by Hamming distance [5]. Only reads presenting Hamming value equal or smaller than the allowed mismatches value are selected for assemblage. Each read mapped generates a list of index values based on the reference genome and the first character of the read. The nucleotides of each position in the mapped genome are choosed taking in count the amount of reads matching it. The most abundant nucleotide is used to the assemblage of the new genome.

The software is freely available in http://www.microbiologia.ufrj.br/ssrna. It is only necessary to install Java virtual machine 7 (free available in http://www.java.com) or higher.

### Sequencing of CLRDV genome by deep-sequencing

Leaves of cotton (*Gossypium hirsutum*) plants (cultivar Fibermax966) infected in green house conditions with Cotton Leafroll Dwarf Virus (CLRDV) by viruliferous aphids inoculation were used for total RNA extraction. The CLRDV virus correspond to a PV1 isolate that suffered more than 5 years of passages in green house conditions at the UFRJ, Rio de Janeiro, RJ, Brazil. Leaves from mock-infected plants were used as negative control. Total RNAs was extracted using the Invisorb Spin Plant RNA Mini Kit (Invisorb®). The quantity and quality of RNA samples obtained were determined by spectrophotometry (Nanodrop ND-1000, Thermo Fisher Scientific) and agarose gel electrophoresis. Systemic infections were confirmed using nested (RT)-PCR assays to detect the viral capsid protein-encoding gene as previously described [6].

The procedures for obtaining the viral small sRNA library was already described [6]. In brief, RNA samples were precipitated in ethanol and sequenced at Fasteris Co. (Geneve, Switzerland) with an Illumina Genome Analyzer (Illumina, San Diego, USA). Small RNAs of 15–30 nt were purified from acrylamide gel; the 3′ IDT miRNA cloning linker (Integrated DNA Technologies, San Diego, USA) and then the 5′ Illumina adapters were single-stranded ligated with T4 RNA ligase to the purified small RNAs. The constructs were purified again on an acrylamide gel to remove empty adapters and then reverse-transcribed and PCR-amplified. The primers used for cDNA synthesis and PCR were designed to insert an index in the 3′ adapter.

The libraries were quality controlled by cloning an aliquot into a TOPO plasmid and capillary sequencing 4–8 clones. High-throughput sequencing was performed on a Genome Analyzer GAIIx for 38 cycles plus 7 cycles to read the indexes. After demultiplexing and adapter removal, 10.5 million pass filter reads were obtained in the library.

The deep sequencing libraries were deposited at GEO (Gene Expression Omnibus) under the number GSE311062 (http://www.ncbi.nlm.nih.gov/geo/info/submission.html).

### Datasets and accession number

*Human immunodeficiency virus* 1 (HIV-1) reads were downloaded from SRA database (http://www.ncbi.nlm.nih.gov/sra) using Aspera software. The accessions numbers of HIV-1 dataset was SRP007924. SPFMV reads were downloaded from https://research.cip.cgiar.org/confluence/display/cpx/CIP.sweetpotato.2008.PER.CIPHQ.siRNA-1.tables.

The accessions numbers of the complete genome virus used in this paper were: HIV - NC_001802.1; *Human herpesvirus* 1 (HHV-1) - NC_001806.1; Sweet potato feathery mottle virus (SPFMV) - NC_001841.1; Sweet potato feathery mottle virus isolate Piu3 (SPFMV – Piu3) - FJ155666; CLRDV-ARG isolate - GU167940; CLRDV-PV1 - HQ827780.1.

All alignments were performed using MultiAlign [7].

## Results

### Software implementation and description

**Figure 1:**
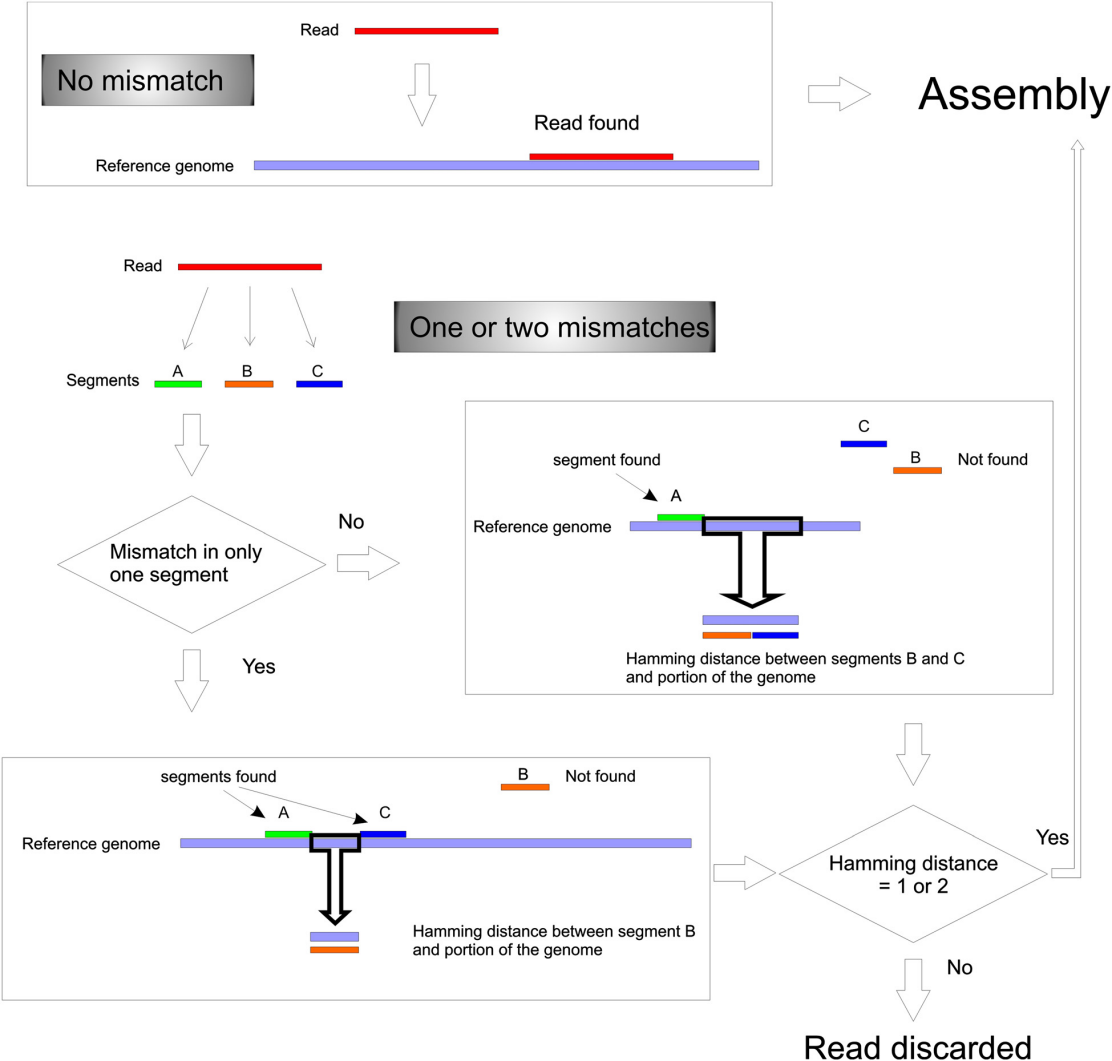
**Reads selection.** Reads are compared to the reference genome. Reads that match 100% with it are selected for further mapping (panel superior). However, reads that do not match 100% the reference genome are divide in three segments and each of them are compared to the reference genome. If one of these segments matches it, it will be compared with the three segments by Hamming distance and selected as positive accordingly to the allowed mismatched previously selected by the user. The user can choose to perform the analysis with no mismatch or allowing up to 3 mismatches to the reference genome.

SearchSmallRNA was created in Java and is publicly released. It has a graphical interface and works on all operating systems. The program performs comparisons between each read sequenced present in a library and a chosen reference genome. The screening uses a comparing strings method, which returns an index value when a read matches perfectly to a specific region of the reference genome (Figure 1). Non-matching reads (Figure 1B) are divided into three segments and each of them is compared to the reference genome. If one of these divided segments returns a positive index value, it will be compared with the three segments by Hamming distance [5]. Reads showing Hamming distances smaller or equal to the allowed mismatched previously selected by the user will be selected as positives.

All the recovered reads are then assembled generating a long nucleotide sequence. To assemble a unique uninterrupted genomic strand, the regions of the reference genome not covered by sRNA reads are filled by dashes.

**Figure 2:**
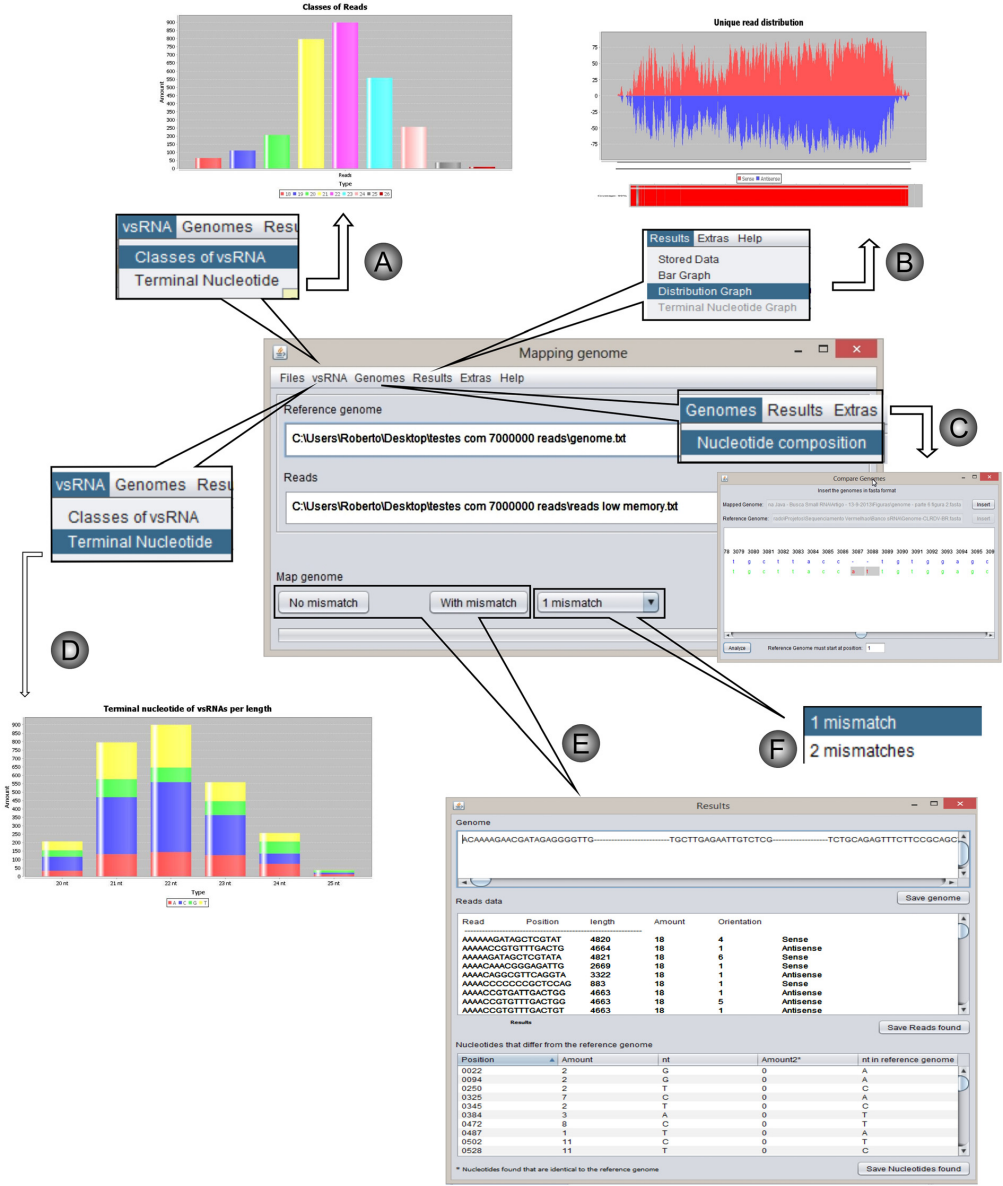
**The SearchSmallRNA platform.** The main window of the program is shown at the center of the figure. Key functions and menus are labeled as **A**-**F**. **A** - Example of a graphical representation of the reads distribution by size. The total numbers of unique reads of all vsRNAs from size classes of 18–26 nts are shown on the X-axis. **B** – Example of the distribution graph of positive reads along the reference genome. Reads matching the genome in the sense orientation are shown in gray and those in the antisense orientation are shown in yellow. The X-axis shows the total number of reads. The red bar below shows where the putative genome generated matches the reference genome; **C** – Nucleotide composition command compares differences between mapped and reference genome. Users can visualize easily gaps and nucleotide divergences that are shown in different colors; **D** – Example of a graphical analysis of the terminal nucleotide from the positive reads/vsRNAs. **E** – Result windows showing the details of each read mapped; and **F** – A combination box that allows the user to choose how many mismatches between the read and the reference genome will be allowed in the analyses.

The genomic strand generated, including the details of each read in terms of position, amount, length and orientation are visualized on the computer screen after analysis. Figure 2 shows an overview of the program features. The program can also create three different types of graphics and tables with relevant read information, providing a profile of the nature, size and numbers of vsRNAs (see Figure 2A-F). It also provides an analysis of the terminal nucleotides of the reads, which may provide clues to vsRNA biosynthesis.

SearchSmallRNA can works on computer with low memory resources; therefore, mapping analysis may be performed on personal computers in a few minutes.

### Software validation

To validate the program, libraries of small RNAs from the *Human immunodeficiency virus* 1 (HIV-1), family Retroviridae, and two plant viruses (the *Sweet potato feathery mottle virus* (SPFMV) and the Cotton leafroll dwarf virus (CLRDV)) from families Potyviridae and Luteoviridae, respectively, which were sequenced by deep-sequencing, were mapped to their respective genomes.

Using HIV-1 (NC_001802.1) as the reference genome and the dataset of HIV-1 from SRA (SRP007924), we were able to reconstruct the HIV-1 genome with the software. The coverage of the generated genome sequence was 86% when one mismatch was allowed and 92% when allowing two mismatches (data not shown). Despite the presence of some gaps, the genomic sequence obtained covered almost the entire genome. Long nucleotides sequences of approximately 1,450 base pairs with no gaps were recovered.

In order to check the reliability of the software analysis, the *Human herpes virus* – 1 (HHV-1 or HSV-1) genome was used as a negative control reference genome for mapping the reads obtained for *Human immunodeficiency virus* - 1, Retroviridae family sequencing. No reads of this library mapped against the HSV genome (data not shown).

**Figure 3:**
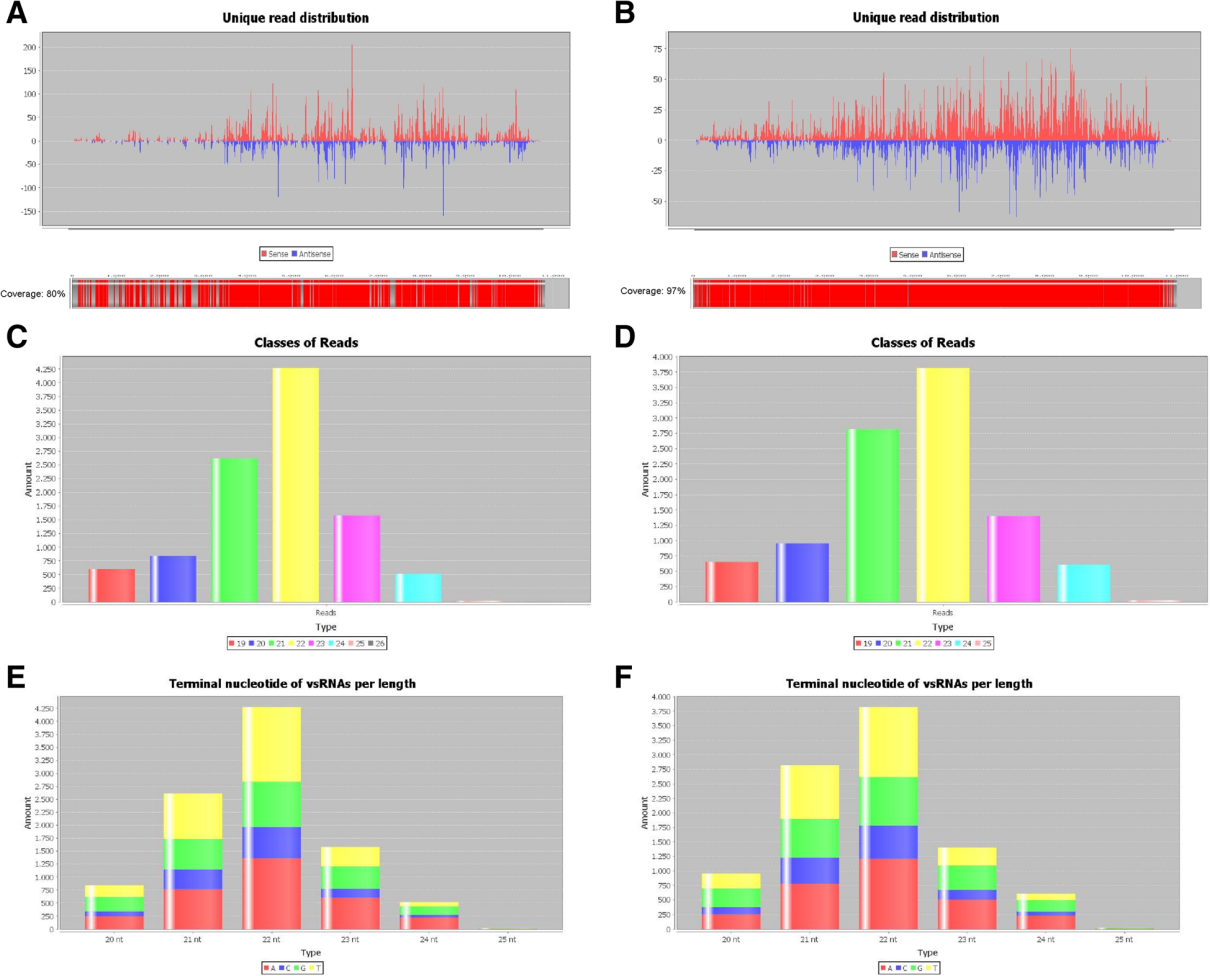
**SearchSmallRNA results using the SPFMV-Piu3 small RNA library dataset against two distinct reference genomes SPFMV-Piu isolate (FJ155666) and SPFMV (NC_001841.1). A** and **B** show a graphical view of the read distribution obtained after the screening using SPFMV genome and 2 mismatches and SPFMV Piu3 genome without mismatch. **C** and **E** and **D** and **F** show the size distribution of the reads and their distribution considering its 5′ terminal nucleotides, using SPFMV and SPFMV-Piu 3, respectively. X-axis shows the total number of reads.

In the analysis of SPFMV, two available references genomes were used: one obtained by the Sanger sequencing method (NC_001841.1) [8] and the other, the SPFMV Piu3 isolate (FJ155666) by NGS of sRNAs [2]. The dataset of the vsRNA obtained by Kreuze and co-authors where used for the genome reconstruction. Figure 3 shows the read distribution obtained by the SearchSmallRNA software along each reference genome. Using NC_001841.1 as the reference genome, the software-generated genome achieved 61% of correspondence to it when allowing two mismatches in the search (Figure 3A). Alignment between SPFMV Piu3 and SPFMV NC_001841 isolates shows 87.4% of similarity at the nucleotide level, so this relatively small correspondence was expected. Mapping using the Piu 3 dataset with the Piu3 isolate as reference genome, on the other hand, generated a genome sequence corresponding to 98% of the reference genome when a single mismatch was allowed and to 97%, when no mismatches was selected, as expected (Figure 3B).

Viral read distribution by size was obtained for both isolates (Figure 3C and D). Independently of the reference genome used, most part of the vsRNAs have 22 nucleotides in size, as already observed for other viruses [9]. The distribution of 5′ terminal nucleotides was also determined for the vsRNAs identified in each case (Figure 3D and E). Similar patterns were observed for all the four types of SPFMV-vsRNAs characterized (i.e., 21-, 22-, 23- and 24-nt) in both cases. Timidine was the most commonly found nucleotide at the 5′ terminus for vs-RNA with 21- and 22-nt.

**Figure 4:**
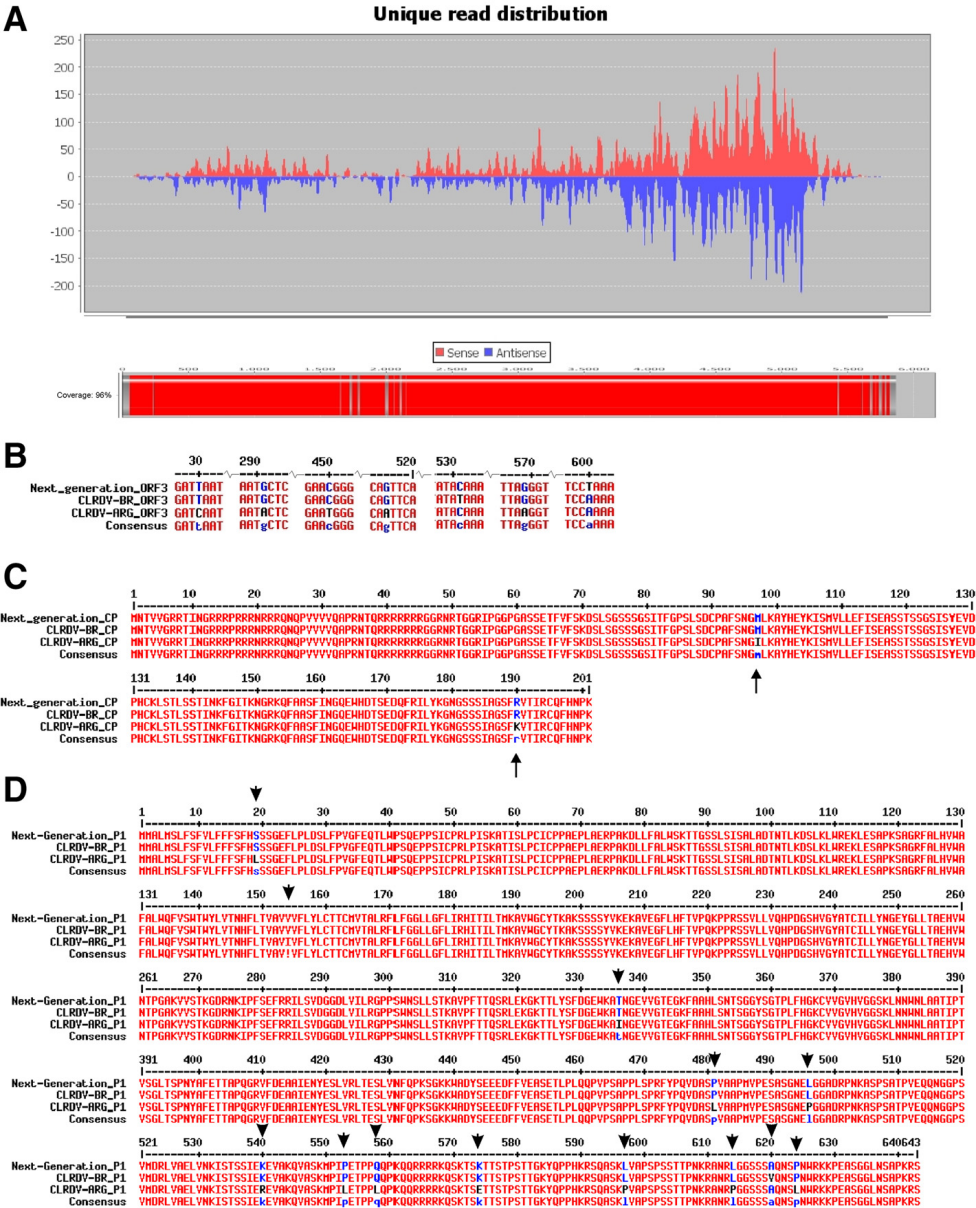
**CLRDV mapping. A** – Graphical of viral unique reads distribution of CLRDV-infected leaves smallRNA dataset using the genome of CLRDV-ARG as reference genome. In gray reads in sense orientation are shown and in yellow the reads showing antisense orientation. Red bar at bottom shows the total of mapped genomes and white block represents gaps not covered by the analysis. X-axis is showing the total number of reads. **B** – Details of divergent points in ORF3 (CP) nucleotide alignment. **C** – Capsid protein (ORF3) amino acids alignment. **D** – P1 protein amino acids alignment. Arrows are indicating divergent amino acids. Next_generation corresponds to the genome reconstructed, CLRDV PV1 and CLRDV ARG corresponds to Brazilian and Argentinean isolates, respectively.

Using an infected cotton vsRNA library from our group [9], we were able to assembly the CLRDV genome. Using the CLRDV-ARG isolate (GU167940) as the reference genome, an almost complete viral genome sequence was achieved as we can observe in Figure 4A in the bottom rectangle colored in red. To fill the remaining gaps present on the reconstructed sequence, RT-PCR primers were designed and the resulting amplicon sequenced by Sanger method (data not shown). Amplicons sequences corresponding to assembled regions without gaps showed that the genome sequence generated by SearchSmallRNA has a high degree of reliability. The reconstructed sequence was very similar to the two available CLRDV complete genomes, the isolate ARG, from Argentine, and isolate PV1, from Brazil.

In order to check the reliability of the reconstructed sequence, called here “next_generation or NGS”, we aligned it with CLRDV ARG and PV1 genomes. The reconstructed genome was almost identical to the CLRDV PV1 isolate. Two examples are shown in Additional file 1: Figure S1 and Figure 4B-D where we can see the alignment of nucleotides and amino-acid sequences, respectively, of two important viral proteins, polymerase or P1 and coat protein, or CP. Seven divergent nucleotides were found between the 606 nucleotides of the complete ORF3 (CP) at positions 30, 291, 450, 516, 531, 569 and 600, respectively (Additional file 1: Figure S1 and Figure 4B). However, between these 7 divergent nucleotides, 5 nucleotides were similar between the NGS sequence and the CLRDV PV1, as can be seen at position 30, where the two sequences have a T; at positions 291, 516, 569 where they share a G; and at position 450, where they have a C. One divergent nucleotide matches the CLRDV ARG (a C at position 531, instead of a T at CLRDV PV1) and one do not match any of the two the isolates, as NGS sequence has a T instead of a G as CLRDV-ARG and CLRDV PV1. Thus, the reconstructed nucleotide sequence matches preferentially the Brazilian isolate of CLRDV.

The alignment of the amino acid sequences of these two ORFs confirms these results. Looking the only two divergent amino acids observed in the whole CP sequence at positions 97 and 190, respectively, we can see that the deduced amino acids sequence of NGS matches 100% the CLRDV PV1 isolate (Figure 4C).

Alignment of the P1 nucleotide and amino acids sequences showed similar results. As expected, the number of divergent nucleotides and/or amino acids are higher in this protein sequence as it is not so conserved as CP protein. Among the 33 divergent nucleotides found between the three aligned sequences, 20 were similar between NGS genome and CLRDV PV1 isolate; 9 between NGS and CLRDV ARG, and 4 were “new” nucleotides, not matching neither CLRDV ARG nor PV1 isolates (Additional file 1: Figure S1). At protein level, however, just 13 divergent amino acids were found, indicating that most part of the divergent nucleotides were silent mutations. From these 13 divergent amino acids, 12 are shared by NGS and CLRDV PV1 and one is shared by NGS and ARG isolate. So, as expected, NGS sequence is closely related to PV1 isolate.

Taken together, all these results are indicating the credibility and robustness of vsRNA mapping and assemblage by SearchSmallRNA.

## Discussion

At the current rate of technological progress, high-throughput sequencing of nucleic acids has become a commodity. These techniques are perfectly suitable for viral small RNA sequencing and contribute to the understanding of many aspects of virus biology in the context of host-pathogen interaction. However, the mapping of the viral genomes using total small RNA dataset is still an issue and remains a challenge that has to be faced by bioinformatic experts. Even with these limitations, new viral and viroids’ genomes are now been published using small RNA sequencing from infected cells and/or tissues [2,3,10,11]. Here we show the development free user-friendly software with a graphical interface that enables researchers with no bioinformatic expertise to analyze theirs own dataset or library of total small RNAs from virus infected cells/organisms.

SearchSmallRNA shown to be able to recover almost complete viral genomes using the full dataset library of small RNA sequencing by NGS, with no need of previous steps of *in silico* subtraction of host genomes or others before mapping [10,11]. Just the sequencing adapters should be removed. As this program do not make assembly, a limitation of its use could be the necessity of a reference genome to be able to recover the information concerning the virus in study from the dataset. So, to start the analysis, it is important to have previous ideas or hypothesis about the infecting virus in study, as its genera or species. Although, even using a relative distant virus to start the mapping, it can reconstruct partial genomes from unknown viruses that can be used to start to design primers to further amplify its genome.

The software efficacy and reliability was tested by different ways. Using web available small RNA and RNAseq datasets we showed that the software could reconstruct the viral genomes with high levels of confidence.

Using the whole library of infected CLRDV cotton plants, the genome sequence reconstructed by SearchSmallRNA confirms it ability to reconstruct reliable genomes. As we could observe analyzing the alignment of viral proteins between the reconstructed genome given by the program, called next_generation or NGS, and two complete genomes of this virus, the NGS sequence was more similar to it closest isolate, PV1. Furthermore, nucleotides divergent from both, Brazilian and Argentine, genomes were found in the NGS sequence showing that in fact, even using a more distant reference genome to start the analysis we can recovery the correct genome. So small differences in the reference genome does not alter the sequence reconstructed. Even “new” divergent nucleotides, not previously observed in the already described genomes could be observed. Therefore the validation experiments showed that the viral genomes assembled using the program described in this paper are quite reliable and robust.

## Conclusions

SearchSmallRNA has shown to be reliable and robust software that can be used to help the biologists to map and assemblage of new virus isolates using NGS data. Using its easy and friendly graphical interface, researchers will be able to obtain viral genome maps corresponding to the virus in study in few minutes. After obtain this partial/incomplete map, however, PCR or RT-PCR followed by Sanger sequence will be necessary to complete gaps of no matching regions. Even with the necessity of this further step, common for other existing map program, the use of the program will accelerate and simplify consistently the mapping using small RNAs libraries obtained by NGS, dispensing the need of bioinformatics experts and/or the buy of expensive mapping software.

### Availability of supporting data

SearchSmallRNA supporting data are available at http://www.microbiologia.ufrj.br/ssrna/.

## Competing interests

The authors declare that they have no competing interests.

## Authors’ contributions

RRSA design and developed the software, performed all the validations analysis and drafted the first version of manuscript. MFSV conceived the study, coordinated all the analysis and draft the manuscript. Both authors read and approved the final manuscript.

## Supplementary Material

Additional file 1: Figure S1 Nucleotide alignment between the mapped sequence, next_generation, the CLRDV-BR and the CLRDV-ARG. A shows ORF3 or CP and B, ORF1 or P1, alignments, respectively. Click here for file
