# Supplementary figures and images for: SearchSmallRNA: a graphical interface tool for the assemblage of viral genomes using small RNA libraries data
Source: Virol J. 2014 Mar 7;11:45. doi: 10.1186/1743-422X-11-45 (PMC4007622; doi:10.1186/1743-422X-11-45)

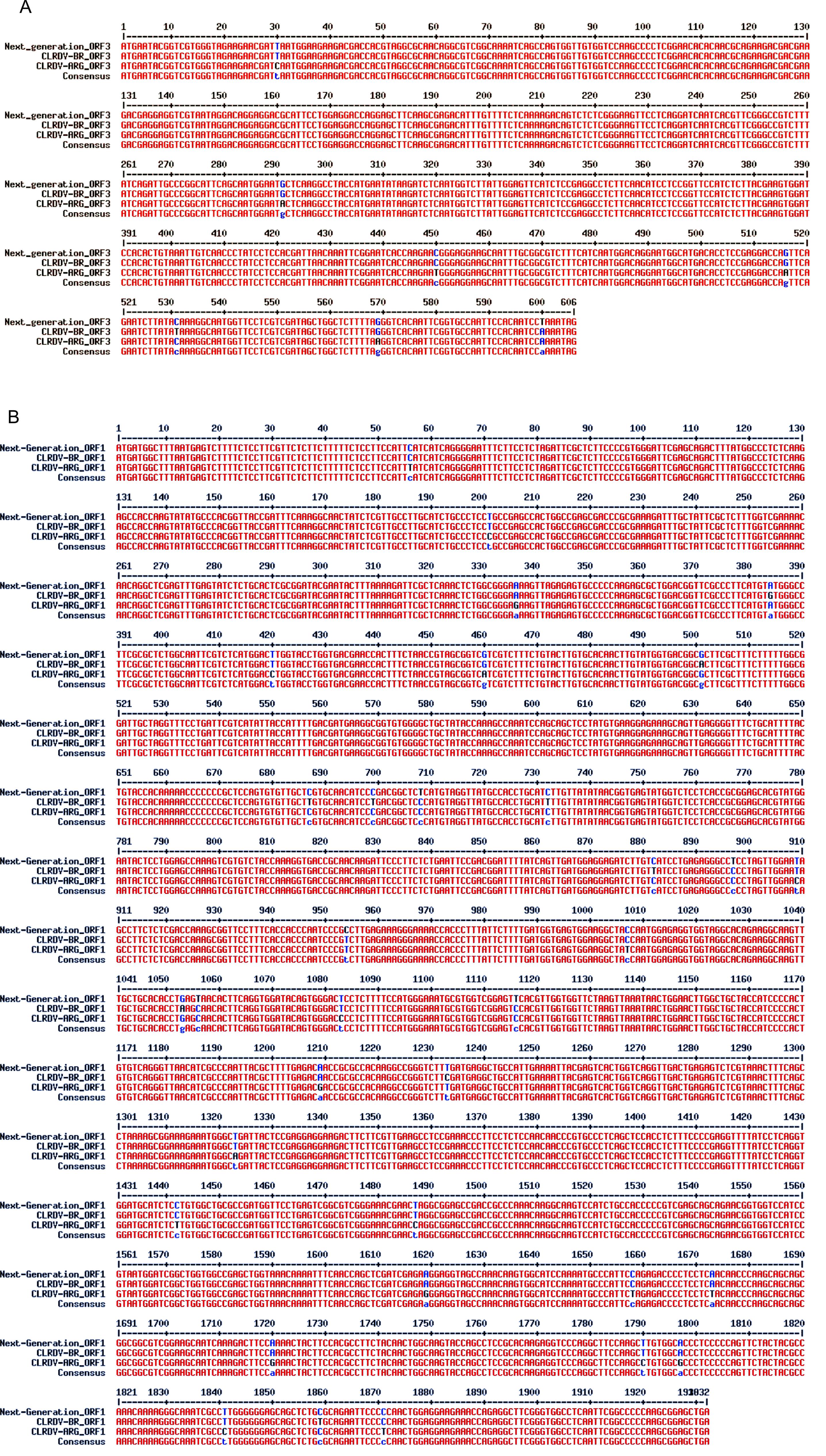

Supplement: Additional file 1: Figure S1 — Nucleotide alignment between the mapped sequence, next_generation, the CLRDV-BR and the CLRDV-ARG. A shows ORF3 or CP and B, ORF1 or P1, alignments, respectively. [file 1743-422X-11-45-S1.jpeg]
